# Prevention of food and airway allergy: consensus of the Italian Society of Preventive and Social Paediatrics, the Italian Society of Paediatric Allergy and Immunology, and Italian Society of Pediatrics

**DOI:** 10.1186/s40413-016-0111-6

**Published:** 2016-08-18

**Authors:** Giuseppe di Mauro, Roberto Bernardini, Salvatore Barberi, Annalisa Capuano, Antonio Correra, Gian Luigi de’ Angelis, Iride Dello Iacono, Maurizio de Martino, Daniele Ghiglioni, Dora Di Mauro, Marcello Giovannini, Massimo Landi, Gian Luigi Marseglia, Alberto Martelli, Vito Leonardo Miniello, Diego Peroni, Lucilla Ricottini Maria Giuseppa Sullo, Luigi Terracciano, Cristina Vascone, Elvira Verduci, Maria Carmen Verga, Elena Chiappini

**Affiliations:** Department of Sciences for Health Sciences, Anna Meyer Children’s University Hospital, University of Florence, Viale Pieraccini, 24, Florence, 50100 Italy

**Keywords:** Allergy, Children, Prevention, Consensus

## Abstract

**Background:**

Allergic sensitization in children and allergic diseases arising therefrom are increasing for decades.

Several interventions, functional foods, pro- and prebiotics, vitamins are proposed for the prevention of allergies and they can’t be uncritically adopted.

**Objective:**

This Consensus document was developed by the Italian Society of Preventive and Social Paediatrics and the Italian Society of Paediatric Allergy and Immunology.

The aim is to provide updated recommendations regarding allergy prevention in children.

**Methods:**

The document has been issued by a multidisciplinary expert panel and it is intended to be mainly directed to primary care paediatricians.

It includes 19 questions which have been preliminarily considered relevant by the panel. Relatively to each question, a literature search has been performed, according to the Italian National Guideline Program. Methodology, and a brief summary of the available literature data, has been provided.

Many topics have been analyzed including the role of mother’s diet restriction, use of breast/formula/hydrolyzed milk; timing of introduction of complementary foods, role (if any) of probiotics, prebiotics, vitamins, exposure to dust mites, animals and to tobacco smoke.

**Results:**

Some preventive interventions have a strong level of recommendation. (e.g., the dehumidifier to reduce exposure to mite allergens). With regard to other types of intervention, such as the use of partially and extensively hydrolyzed formulas, the document underlines the lack of evidence of effectiveness.

No preventive effect of dietary supplementation with polyunsaturated fatty acids, vitamins or minerals has been demonstrated.

There is no preventive effect of probiotics on asthma, rhinitis and allergic diseases. It has demonstrated a modest effect, but steady, in the prevention of atopic dermatitis.

**Conclusions:**

The recommendations of the Consensus are based on a careful analysis of the evidence available.

The lack of evidence of efficacy does not necessarily imply that some interventions may not be effective, but currently they can’t be recommended.

## Background

A peak in allergic, especially respiratory, disease prevalence has been recently reported in Western countries [1–5]. As an example, a multicenter study conducted in the Italy, as part of the European Community Respiratory Health Survey (ECRHS), reported a rhinitis prevalence of 18.5 % among children, with an increase of more than 50 % over the previous decades [6]. Bronchial asthma prevalence has increased, as well, as reported by The International Study of Allergy and Asthma in Childhood (ISFAC) [7].

Rhinitis and asthma affect both the quality of life in relation to health (as measured by Health-Related Quality Of Life (HRQOL) and the cost of treatment. Several studies show that rhinitis has negative effects on the activities of the patient’s everyday life at home, school and work [8, 9]. The questionnaire designed to measure the effect of rhinitis on quality of life (QoL) showed patients with sleep problems, emotional issues and limitations in their activities and social relationships [10]. Similarly, studies have been carried out to assess the QoL of children and adolescents with asthma through the use of specific questionnaires such as the Paediatric Asthma Quality Of Life Questionnaire (PAQLQ), the Adolescent Asthma Quality of Life Questionnaire (FAQLQ) or the Rhinasthma QoL Questionnaire [11, 12]. Studies of the impact of asthma on QoL have reported that asthma causes greater limitation of physical activity and has greater influence over the emotional sphere in girls while the PAQLQ score decreases with the progression of disease severity [13, 14]. In contrast, a recent meta-analysis of 3550 children with asthma demonstrated that many patients were suffering from depression and anxiety disorders, regardless of the level of severity of their disease [15].

In countries such as Australia, the United States and UK, the past decade has been marked by a “second wave” of allergic disease in the form of food allergy **(**FA**)** [1]. Reliable data on this prevalence in other European countries are lacking [16]. “Perceived” FAs are often not confirmed using diagnostic tests. In one study an incidence between 12.4 and 25 % in self-reported FA is estimated, whereas confirmation by oral provocation test (OPT) only occurred in 1.5–3.5 % of cases [17]. In general, FA is more common among children with an incidence of 5–8 %, as compared to 1–2 % in adults [18]. Few epidemiological studies make use of the diagnostic gold standard, the double-blind, placebo-controlled food challenge. Thus, more research is needed for a more accurate determination of the prevalence and incidence of FA in the paediatric population [16]. FA represents a significant problem in terms of morbidity and mortality if one considers that foods are the most important trigger of anaphylaxis in children, although the prevalence of fatal anaphylaxis is 0.001 % [19].

The results of 10 studies from Europe suggest an incidence of anaphylaxis ranging between 1.5 and 7.9/100,000/year [19]. Studies conducted in the UK reveal an increase in hospital admissions for anaphylaxis over the last two decades [19]. Three European studies show a prevalence of anaphylaxis of 0.3 % (95 % CI: 0.1–0.5 %) [19]. In the last 10 years, studies to assess the impact of FA on the quality of life of patients have been published. The disease has a significant effect on the quality of life of children and their families and on healthcare costs, both in numbers of outpatient visits and repeated admission to emergency departments. Moreover, elimination diets can trigger authentic food phobias among parents, and sometimes consequent eating disorders, especially if the disease is severe, as it is in particularly sensitive patients at risk of anaphylaxis even with small ingested doses [20, 21].

For all these reasons, many studies have evaluated the possibility of primary prevention of allergic diseases for children at high risk of developing them. The objective of the present Consensus is to define the evidence regarding the actual impact that environmental, behavioural and nutritional preventive measures may exert on the incidence and prevalence of respiratory and food allergies.

## Methods

In the present document a children at risk of allergic disease has been defined as any child with at least one first-degree relative (parent or sibling) suffering from atopic disease.

*Search strategy*

Using the Consensus Conference method based on the National Institutes of Health and the Italian National Programme Guidelines (Piano Nazionale Linee Guida, PNLG), relevant publications in English were identified by a systematic review of MEDLINE and the Cochrane Database of Systematic Reviews from their inception until December 31, 2014. The search strategy was “children [Title/Abstract] OR pediatric [Title/Abstract] AND allergy prevention [Title/Abstract] OR prevention [Title/Abstract] OR allergy [Title/Abstract] AND asthma [Title/Abstract] OR rhinitis [Title/Abstract] or food allergy drug [Title/Abstract] AND English [lang]).” The Working Group agreed on a list of clinical questions (listed below) and provided specific additional search strategies for each questions.

The analysis and evaluation of the Guidelines was made according to the following minimum criteria of validity: multidisciplinary panel, systematic search for evidence, grading of recommendations.

Analysis of Systematic Reviews, Randomized Controlled Trials and Observational Studies were made using the AMSTAR (Assessment of Multiple Systematic Reviews) tool [22], the Cochrane Risk of Bias Tool [23] and the Newcastle Ottawa Scale [24] respectively.

**Question 1. Should exclusive breastfeeding be promoted against infant formula (formula) for the prevention of allergic diseases in high risk infants?**

**Introduction**

Breastfeeding is associated with several beneficial effects on mother and child health and is therefore recommended for all infants [25]. Several potential, mechanisms may be linked to the prevention of allergic disease through exclusive breastfeeding, such as limited exposure to exogenous antigens, protection against infections, promotion of the maturation of the gastrointestinal mucosa, development of “beneficial” gut microbiota and carrier substances for immunomodulatory and anti-inflammatory actions (e.g. n-3 LCPUFA) [26].

The association between breastfeeding and the prevention of allergic disease has been frequently studied and has often been debated in the last 70 years. Some studies have shown a protective effect, others failed to show any effect and others still have demonstrated a predisposing effect. Despite conflicting and controversial data in the literature, this should not be interpreted as meaning that breastfeeding has no noticeable effect. In fact, the association between breastfeeding and prevention of allergic disease remains inadequately studied and their interactions are complex. The existing randomised, double-blinded studies cannot be included in systematic reviews and meta-analyses and most published studies are retrospective. Furthermore, the definition of breastfeeding itself is imprecise [27].

In particular, many studies do not distinguish between exclusive and non-exclusive breastfeeding. Moreover, the nomenclature to define allergic outcomes is often used incorrectly (wheezing and asthma). Finally, the investigation of the association between breastfeeding and allergy prevention can be complicated by the presence of polymorphisms in the fatty acid desaturase (*FADS*) gene cluster. The resulting enzymatic activity leads to the production of long chain fatty acids (LCPUFA) and influences the levels of LCPUFA in mother’s milk, thereby modulating the association [28].

In 1988, Kramer developed 12 standards for the design and analysis of studies assessing the association between breastfeeding and allergic disease. These included prospective design, sufficient duration of exclusive breastfeeding, specific definition of allergy outcomes for analysis, the evaluation of effects on the at-risk population and ensuring adequate statistical power [29]. To date, however, no published study has met these criteria, and both the association and the controversy between breastfeeding and allergy remain unresolved.

**Summary of literature data**

Effect on allergic rhinitis

A meta-analysis of prospective studies found a protective effect of exclusive breastfeeding of 3 months’ duration or more to be close to statistical significance in the general population (OR 0.74; 95 % CI) 0.54–1.01 but not in children with a family history of atopic disease (OR 0.87; 0.48–1.58 95 % CI) [30]. Of the later studies conducted in this field, only one appears to be prospective and observed reduced risk of allergic rhinitis (OR 0.8; 0.6–0.9 95 % CI) at 3 years of life in an African-American paediatric population at risk [31].

Effect on wheezing and asthma

Breastfeeding exclusively for the first 3–4 months of life appears to be associated with a reduction in episodes of wheezing caused by an upper airway infection in the first 4 years of life [32]. Wheezing episodes after 6 years may be symptoms of allergic asthma. In this setting, the results of the study on a protective effect of breastfeeding remain controversial [32].

Two meta-analyses of prospective studies also reported controversial results regarding the protective effect of 3 or more months of exclusive breastfeeding against the risk of developing asthma in individual children at risk of atopy [33, 34]. Some studies even suggest that exclusive breastfeeding for 3 months increases the incidence of asthma after 14 years of age in children with atopic risk factors [32].

Recently, a study has shown that the prevalence of asthma in 10 years of life is reduced only in populations of children who were exclusively breastfed for at least 3 months and who were carriers of at least one minor allele polymorphism in the *FADS* gene cluster [28]. Children homozygotes for the major allele failed to benefit from exclusive breastfeeding altogether [28].

Effect on food allergy

There is insufficient evidence to conclude about a benefit of breastfeeding in the prevention of food allergy in infants with an atopic risk [35–37]. A multidisciplinary review, however, concluded that breastfeeding seems to exert a protective effect, particularly amid children at risk [38]. A prospective, randomised clinical trial in preterm infants reported a lower cumulative incidence of cow’s milk protein allergy (not food allergy in general) 18 months after term. This marked reduction especially concerned events such as allergic eczema. Infants who had been fed with breast milk from a human milk bank for more than 4 months performed better that their peers on a diet of term and preterm infant formula [39]. In a more recent systematic review two cohort studies showed, that exclusive breastfeeding had no beneficial effect or was associated with an increased risk for allergy in the population at risk for atopic disease [37, 40, 41]. Wetzig et al. showed that exclusive breastfeeding for 5 months or longer was associated with greater egg sensitisation in 1 year, but did not include any data about food allergy [41].

Effect on atopic dermatitis

In one meta-analysis, exclusive breastfeeding for 3 months rather than formula was associated with a greater reduction in the incidence of atopic dermatitis [42, 43]. However, a subsequent systematic review and meta-analysis found no effect of breastfeeding, and removed one controversial study from the analysis [25].

**Box 1. Breastfeeding definition**

**Table:** 

Exclusive breastfeeding: The infant who receives only human milk (including donor milk). Oral rehydration solution (ORS), syrups (vitamins, minerals) and medicines are allowed but nothing else. Predominant breastfeeding: the predominant source of nourishment is breast milk. Water and water-based drinks, oral rehydration salts solution, drops and syrups (vitamins, minerals, medicines) are allowed but nothing else. Infant formula food alone can satisfy the nutritional requirements of the first 6 months of life (Directive 2006/141/EC implemented by Ministerial Decree n. 82 of 9th April 2009). Follow-on formula: food constituting the principal liquid element of the diet of infants, after diversification at 6 months of age (Directive 2006/141/EC, implemented by Ministerial Decree n. 82 of 9th April 2009).	

In conclusion, although the data from the literature remain controversial and no single protective effect of breastfeeding can be shown to be effective against the onset of allergic disease, an exclusive breastfeeding regimen for 6 months should be promoted in view of the known and recognized nutritional and immunological benefits of breast milk.

**Recommendation. Exclusive breastfeeding (possibly for 6 months and for at least 4 months) should be promoted for the known and recognized nutritional and immunological benefits of breast milk.**

**Weaning**

**Question 2. Is weaning between the 4th and 6th month of life recommended for the prevention of allergic diseases in children?**

**Introduction**

The World Health Organization recommends exclusive breastfeeding for the first 6 months of life [44]. The American Academy of Pediatrics recommends the introduction of “complementary foods” no earlier than 4 months and recommends exclusive breastfeeding as indicated for up to 6 months [45]. Early exposure to solid foods (<4 months) has been blamed for the development of allergic disease in the past and especially among patients with atopic dermatitis. A paradigm shift is currently under way with the spread of oral tolerance induction as a concept in allergy therapy. Studying the introduction of “complementary foods” after at least 4 months of exclusive breastfeeding may lead to a reduction in the prevalence of food allergy.

**Knowledge-based approaches**

Several cohort studies, such as GINI, LISA or KOALA have failed to capture an effect of delaying the introduction of solid foods in terms of food allergy prevalence [46–48]. The strategy of delayed solid food introduction until the 6th month does not seem to confer prevention benefits, as shown in two cohort studies the quality of evidence of which is rated as low [49, 50].

Introduction of potentially allergenic foods (cow’s milk, eggs, fish, nuts)

There are two randomised, controlled clinical trials that show that early exposure to cow’s milk protein in the first few days of life is not associated with an increased risk of food allergy [51–53].

However, in one of these studies the diagnostic criteria for food allergy do not include confirmation by OFC (Oral Food Challenge) and, in the other, the symptoms are unspecific and food allergy data are not reported. Another randomised trial and a cohort study have shown an increased risk of cow's milk protein allergy with neonatal exposure to these proteins, especially among children at risk of atopy [51–55].

In a prospective observational cohort study of 13,019 children, the relative risk of those exposed to the CMP in the first 15 days of life than those exposed after 15 days = 0.045 (in practice it would be about 20 times less likely to develop a CMP sensitization if their introduction is done before 15 days of life) [56]. About the introduction of hen’s egg, one observational study reported an increased risk of allergy with more delayed introduction (>9 months) even after adjusting for confounding factors [57]. Regarding the fish and egg introduction, current international nutritional recommendations for the general population do not suggest delayed introduction (>9 months) but actively indicate exposure within a short time of the introduction of solid foods, preferably while the infant is breastfed [58, 59]. This is an important point for emphasis from an allergy as well as nutritional point of view [60]. In fact, the introduction of such foods, along with breastfeeding, can maintain the profile of the most beneficial nutrient intake, since associated with a lower intake of protein, the greater contribution of LCPUFA, especially DHA, respect to only meat and cheese [60]. The recently published results of two RCTs (LEAP [61] and EAT study [62]) would seem to prove the preventive efficacy of early exposure to allergenic foods (peanuts, fish, CMP, egg, sesame). In fact, in the LEAP study, the “early ” exposure was between 4 and 11 months and the EAT study is burdened by many biases including: the very low compliance to intervention (just 30–40 %), diagnostic confirmation with OFC 6–12 months after allergic reactions, non-registration of allergy cases between 3 and 6 months.

**Box 2: Definition of “complementary foods”.**

**Table:** 

“Complementary foods”: all liquid, semi-solid and solid foods which differ from breast milk and formula milk. World Health Organization. Indicators for assessing infant and young child feeding practices Part 1: Definitions. Geneva: World Health Organization, 2008, Available from: http://whqlibdoc.who.int/publications/2008/9789241596664_eng.pdf	

In conclusion, available scientific evidence does not allow making specific recommendations about the timing of the introduction of complementary foods for the prevention of allergic disease. Regarding the timing of introduction of potentially allergenic foods, once “complementary foods” have been introduced into the diet, current evidence does not justify either delaying or encouraging exposure without reference to individual atopic risk.

**RecommendationThe introduction of “complementary foods” is recommended after the 4th month and if possible after the sixth, regardless of the mode of feeding and atopic risk.**

**RecommendationOnce feeding “complementary foods” has been initiated, it is recommended to introduce potentially allergenic foods in the same way in the diet of children with or without an allergic risk.**

**Partially hydrolysed formula (pHF), extensively hydrolysed formula (eHF) and functional foods**

**Question 3. In children at risk, in case of hypo/agalattia maternal, you must do prevention with formulas Partially hydrolyzed (partially Hydrolyzed Formulas - PHF) vs infant formula (Formula) to prevent the allergic disease?**

**Question 4. In children at risk, in case of hypo/maternal agalattia, you have to make prevention with extensively hydrolyzed formula (extensively Hydrolyzed Formulas - eHF) vs infant formula (Formula) to prevent the allergic disease?**

**Introduction**

The aim of the present section is to define recommendations for the use of partially hydrolysed formula (PHF) and extensively hydrolysed (eHF) formulas and of some functional foods (ω3 and ω6 polyunsaturated fatty acids, vitamins and minerals) for the primary prevention of allergy. For this purpose, a preliminary evaluation of the available scientific evidence on the safety and efficacy data of the interventions was carried out.

NB: *The composition of the formulas available in Italy under the designation of “hypoallergenic” or HA corresponds to partially hydrolysed milks (“PHF” in this Consensus document)*.

**Partially hydrolysed (PHF) and extensively hydrolysed (eHF) formula**

Exposure to allergens early in life during pregnancy or in infanthood is critical for the development of allergies. Scientific research has therefore focused on children’s diets which involve early exposure to allergens and can easily be modified. Preventive measures tested for allergies and food allergy in particular, have included maternal allergen avoidance during pregnancy and/or breastfeeding, exclusive breastfeeding for a more or less prolonged period and avoidance of potential allergens—both dietary and environmental—during the first year of life and beyond. Allergy is a specific reaction to a normally harmless protein (allergen).

Partial and extensive hydrolysates are manufactured by modifying the allergenic protein content of milk in order to prevent sensitisation. These formulas can be derived from cow’s milk proteins (serum protein or casein) and soy and they are produced by processes of partial or extensive enzymatic digestion that can break the native proteins into peptides of different sizes [63]. The formulas derived from cow’s milk (CMF) contain whole proteins ranging in size from 14 kD (α-lactalbumin) to 67 kD (serum albumin) [64]. There is no agreement about defining a formula as partially, in contrast to extensively, hydrolysed according to the size of its constituent peptides. By convention, eHF contains only peptides ≤3 kD, while PHF contains only peptides ≤5 kD. In practice, however, in both PHF and eHF peptide size can range widely, with PHF including 18 % of its peptides > 6 kD, while eHF may include up to 5 % of peptides > 3.5 kD [65]. The 10–70 kD (and particularly the 10–40 kD) size range has been directly associated to antigenicity in peptides [66].

**Note on methodology**

Please refer to the relevant section for an outline of the search strategy. The evaluation of the scientific evidence is shown in the Appendix. The population selected for preventive intervention includes by definition children at risk of allergies receiving formula milk in addition to, or in place of, breast milk.

As with other prevention studies, outcomes of interest considered were:

- Allergy (any)
- Food allergy (FA)
- Allergic rhinitis
- Asthma
- Atopic eczema
- Adverse events
- Nutritional status

The latest updates of evidence-based guidelines were consulted:

- NIAID5 (National Institute of Allergy and Infectious Diseases) 2010
- EAACI (European Academy of Allergy and Clinical Immunology) 2013 – alimentary allergy [68]
- ARIA (Allergic Rhinitis and its impact on Asthma) Italian Guideline 2013 – Rhinitis [69]
- GINA (Global Initiative for Asthma) 2012 [70]
- BTS (British Thoracic Society)/SIGN 2012 – Asthma [71]
- SIGN (Scottish Intercollegiate Guidelines Network) 2011 [72]
- ASCIA (Australian Society of Clinical Immunology and Allergy) 2016 [73]
- FAD (American Academy of Dermatology) 2014 – Atopic eczema [74]

The meta-analyses and primary studies not included in systematic reviews because they were published after the closing date of the literature search were analysed only if considered of sufficient methodological quality.

**Previous recommendations**

The guidelines of both the NIAID (2010) and the EAACI (2013) recommended the use of hydrolysates designed for the treatment of food allergy for children at risk, instead of standard infant formula (strength of recommendation B). The 2010 NIAID guidelines specify that cost and availability should be considered as counter-indicative. The 202 BTS/SIGN and GINA guidelines do not recommend hydrolysed formula for prevention and acknowledge only a protective effect in breastfeeding. The 2013 ARIA guidelines do not include recommendations for the prevention of rhinitis. The 2011SIGN, which found limited evidence of a protective effect of eHF when compared to CMF, do not report any specific recommendation for the prevention of atopic dermatitis, while eHF in preference to breast milk is specifically not recommended. Even the 2014 ADF guidelines consider that there is insufficient scientific evidence to recommend specific preventive interventions (dietary or otherwise) for the primary prevention of AD.

**Results**

Results are reported for individual allergic diseases. Three systematic reviews (SR) published between 2009 and 2014 were included for these questions [75–77].

Two SR with meta-analysis evaluated prevention against the development of allergic diseases, as a group and individually [75, 76]. One systematic review without meta-analysis only evaluated FA prevention and included the results of primary studies and SR published before 2009 [77]. It also considered a 2012 US Food and Drug Administration review of the scientific evidence in favour of the qualified health claim of partially hydrolysed whey-protein formula (W-PHF) in regard of risk reduction for atopic dermatitis [78].

Most studies of the use of partially and extensively hydrolysed formulas have low methodological quality in respect of one or more of the following factors: incorrect randomisation procedure, limited sample size, loss to follow-up > 20 %, surrogate or irrelevant clinical outcomes (e.g. sensitisation, atopic dermatitis) and FA diagnosis unconfirmed at OFC.

The few randomised studies with clinically relevant outcomes (and a diagnosis of FA confirmed at OFC) carried out in children at risk showed mixed results. Systematic reviews have shown conflicting data at this regards [67–69]. At any rate, current evidence does not substantiate a preventive effect of PHF formula on the development of food allergy in infants [70, 73, 74].

One study reported a lower risk of developing, specifically, cow’s milk protein allergy (CMPA), but it was conducted in a sample of only 67 children. The assessment of the overall impact of other allergic diseases showed no preventive effect, neither in early childhood nor in later life [79].

Some meta-analyses fail to show a preventive effect on the incidence of atopic dermatitis either in early childhood or later [75]. In other studies, a certain preventive effect is reported but the data should be treated with caution [76]. The number needed to treat is 17 [9–119], i.e. 17 children must be treated, in order to prevent one case of atopic dermatitis. Few studies of rhinitis and asthma prevention have been carried out and these failed to establish a protective effect. The comparison between partially and extensively hydrolysed formulas for the prevention of FA or CMPA apparently favours eHF but the number-needed-to-treat estimates for eHF (NNT = 14) in FA and NNT = 25 in CMPA are fraught with inaccuracies and are difficult to apply to clinical practice settings. All studies concur in denying a preventive effect of soy milk [77, 79]. The higher cost of alternative formula (about double that of CMF) and its inferior palatability are well documented [71, 72, 80, 81]. In a meta-analysis, Osborn included two studies of 46 preterm infants to assess weight gain in relation to the use of hydrolysed formula.

**Overall evaluation**

Recommendations about the primary prevention of allergies cannot be separated from the assessment of the methodological quality of clinical studies and from the clinical relevance of their results, on which some specific criteria exert an influence. For example, the diagnosis of FA requires confirmation with OFC, except in the presence of an anaphylactic reaction. Studies that include diagnosis by “self-report”, or are based on symptoms of allergy and/or sensitisation and allergy, are considered of lower validity. They are often included in systematic reviews and the recommendations of some guidelines (GL) are based on them.

Finally, the conclusions drawn from the evidence must be integrated in a cost-effectiveness analysis, taking into account the following factors:

- absolute effectiveness of preventive interventions;
- health gain expected (clinically relevant outcome, effect magnitude and number needed to treat);
- applicability;
- comparison with alternative interventions, including doing nothing;
- use of resources;
- cost;
- compliance, preferences, willingness to pay (WTP), patient value.

Atopic dermatitis is not attributable to food allergy, except in a small percentage of cases. For this reason, evidence-based medicine (EBM) GL have not recommended hydrolysed formulas, either for therapy or prevention. The use of PHF in the prevention of AD has also been the subject of a “qualified health claim”, a reasoned recommendation from the analysis of scientific evidence of the US Food and Drug Administration (FDA) [78]. The FDA concludes that the evidence in support of a preventive effect of pHF on the development DA in children from 0 to 3 years is very limited. The FDA has issued a statement as a warning to consumers, explaining that pHF are hypoallergenic and should not be administered to children with CMPA because of the risk of serious adverse reactions. This warning statement was considered necessary because the correlation between the use of PHF and a lower risk of developing allergies can mistakenly cause the public to consider these foods appropriate for allergic children.

The most recent GL on rhinitis and asthma and preventive allergy do not include the use of hydrolysed formula among preventive measures and recommend only breastfeeding [70–73]. The evaluation of the efficacy and safety of these preventive interventions do not justify the recommendation.

**Recommendation. A careful analysis of the evidence and a cost/benefit valuation do not currently warrant to characterise partially and extensively hydrolysed formulas as safe an effective for the prevention of allergic disease.**

**Appendix**

**Analysis of the evidence**

Overall preventive effect on allergy

In the meta-analysis of seven studies by Osborn et al. in 2009, a total of 2558 children were included [75]. The RR of treatment is 0.79 [0.66–0.94] and the NNT is 12 [8–20], which means that 12 children on average (within a 8–20 range) should be treated for one to benefit from an effective prevention of allergy.

The meta-analysis by Szajewska and colleagues provides RR incidence and prevalence data for children fed pHF. It includes three studies involving a total of 1281 patients (Vandenplas 1995, Chan 2003 GINI 200819) and stratify the results by age (0–12 months, 0–36 months, 0–5/6 years) [76]. Intention To Treat analyses (ITT) do not show statistically significant (SS) differences in the incidence of allergy among PHF and CMF formula-fed subjects.

Preventive effect on food allergy

Osborn et al.’s meta-analysis examined only one study dealing with the effect of hydrolysed formula in 141 children at risk and reports an increased risk of developing FA in children treated, in contrast to controls. One study involving 67 children at risk shows a preventive effect on CMPA (RR = 0.36 [0.15–0.89], and calculates a NNT of 4 [2–17]). The systematic review by De Silva and co-workers reported two studies arguing against intervention [77]. In two revised meta-analyses (Osborn and Szajewska) and four studies, some of which were already included in systematic reviews (GINI 2008 Vandenplas 1992 Chirico 1997 D'Agata 1996) showed a preventive effect of intervention with pHF.

A preventive effectiveness of eHF is reported by two systematic reviews (van Odijk 2003, Hays, 2005), with two studies (Halken 1993 and 2000, Oldaeus 1997) in favour, and one study against the use of eHF (Mallet 1992). Van Odijk and Hays revised reports are not updated in the bibliography and include studies excluded from subsequent revisions because of bias of various types (non-randomised studies, excessive loss to follow-up, surrogate outcomes, comparisons with soy formulas, etc.).

Preventive effect on allergic rhinitis and asthma

Only Osborn’s systematic review shows an effect on rhinitis and asthma, and includes six studies of 1268 children for asthma and 4 studies of 334 children for rhinitis. Results, stratified by age: preschool asthma preschool age and age were not statistically significant).

Preventive effect on atopic dermatitis

The meta-analysis of by Osborn and colleagues found a preventive effect on atopic dermatitis in five studies and three studies not in support of preventive intervention among a population of 2558 patients, 1928 of whom had been included in the GINI study in 2003.

The results, stratified by age are the following:

- Preschool age: not statistically significant (NSS)
- School age (incidence) NSS
- School age (prevalence) NSS

In Szajewska’s meta-analysis only 3 studies could be included in support of intervention among 1281 children, 1113 of whom had been enrolled in the GINI study in 2008 [81]. The results of the ITT analysis, stratified by age are:

- 0–12 months: RR = 0.58 [0.32–1.04]; NNT = 22 (12–99)
- 0–36 months: RR = 0.71 [0.58–0.88]; NNT = 13 (8–33)
- 0–5, 6 years: RR = 0.80 [0.67–0.97]; NNT = 17 (9–119)

The reported results should be evaluated against the methodological validity of the studies. Studies included in reviews and meta-analyses may not only have been intended to define preventive efficacy and safety but also to comparatively evaluate different types of formulas, and can include children at risk and not at risk and different lengths of supplementation. Generally, studies of the use of partially hydrolysed and extensively hydrolysed formulas have low methodological quality in respect of one or more of the following factors: incorrect randomisation, low sample size, over 20 % loss to follow-up, surrogate outcomes or clinically relevant diagnosis (e.g., sensitisation, atopic dermatitis) unconfirmed by gold-standard tests (e.g., OFC for FA).

In the MACS study, included in De Silva’s review for the evaluation of the effectiveness of soy milk, the results did not support a preventive effect of hydrolysed formula, but cases of atopic disease were detected by telephone survey and not directly confirmed by the authors [81]. As for the comparison between pHF/eHF and CMF and the few randomised studies with clinically relevant outcome (including a diagnosis of FA confirmed with OPT) and conducted on children at risk show conflicting results.

The results of two of the three systematic reviews conducted by methodologically sound criteria (AMSTAR score = 9.11) are contrasting [75, 77]. Some studies included among those “in favour” of the preventive effect, actually reported:

- Clinically irrelevant results (Chirico 1997 only on immunogenicity and allergenicity);
- Or irrelevant (Halken 1993 2000: comparison of efficacy and safety between PHF and eHF);
- Or no statistically significant difference between the group fed hydrolysed formula and the control group (GINI 2003, Oldaeus 1997).

One study showed a lower risk of developing, specifically, to the proteins in cow’s milk allergy (CMA), NNT = 4, but that was conducted on only 67 children (Vandenplas 1992).

The overall incidence of allergic diseases did not show a preventive effect, either in early childhood or in later life. Specifically, the study results are very inaccurate estimates with wide confidence intervals in order to demonstrate a preventive effect on the incidence of atopic dermatitis, both in early childhood [NNT = 27 (15–135)], and in later life [NNT = 23 (11–150)].

Few studies of rhinitis and asthma showed, on average, a preventive effect. The results should be considered cautiously because the small sample size and the imprecision of the estimates.

The most recent studies not included in the revisions analysed the results of the 7-, 10- and 15-year follow-up to the GINI study (published in 2016) the findings of which are based on self-reported diagnoses obtained by a questionnaire, or spirometric parameters non validated for the diagnosis of asthma, from a loss to follow-up well above 20 % (35.5 %): the evaluation must therefore take into account these methodological limitations [82, 83].

ITT analysis shows that cumulative impacts are significantly reduced only in the group that was fed eHF-C, but this effect is mainly due to the incidence of AD and the NNT is 11 [69–96]. The differences observed in the prevalence of allergic manifestations are not, however, statistically significant.

The methodological errors of studies with higher sample size are likely to induce a lively discussion about the validity of these results in the scientific community [83–85].

**Functional foods**

**Question 5. Is the use of functional foods (vs. no intervention) recommended for the prevention of allergic disease in children at risk of allergy?**

**Introduction**

Fatty acids are the constituent ingredients of almost all complex lipids in animal and vegetable fats. The absence or presence of double bonds allows distinguishing saturated from (mono- and poly-) unsaturated fatty acids. Saturated and monounsaturated fats are mainly needed for energy requirements, while polyunsaturated fatty acids (PUFA) and long chain polyunsaturated derivatives, with 20 or more carbon atoms (LCPUFA or LCP) play structural and metabolic functions. PUFA α-linolenic acid (ALA, C18: 3 ω-3) and linoleic acid (LA, C18: 2 ω-6) are nutritionally important and are called essential because the body cannot synthesise them and they must therefore be introduced preformed in food. The increased prevalence of atopic disease in industrialized countries in recent decades has been attributed to changes in nutritional habits and, in particular, to fat consumption. According to this hypothesis, the increased intake of linoleic acid, with the consequent increase in the synthesis of arachidonic acid, would lead to increased formation of PGE2, of which arachidonic acid is a precursor. PGE2 is a potent activator of Th2 lymphocytes and leads to a reduction in IFNγ levels and an increase in IL-4. These changes stimulate a B lymphocytes response with the production of IgE which may determine predisposition to allergic sensitisation. Increased intakes of ω-3 in the diet have been associated with a decrease in the pro-inflammatory effects of arachidonic acid at several levels, reducing the production of eicosanoids and a Th2 response.

**Methodological note**

The search strategy is shown in the relevant section. The evaluation of the scientific evidence is made explicit in the text.

The population likely to benefit from preventive interventions includes: pregnant women and lactating mothers of children with a first-degree blood relative of who suffers from atopy, children at risk of allergy, according to the definition provided, taking functional foods as supplements. The latest updates of evidence-based studies, as well as of research and evaluation of evidence, are described in the Appendix.

Previous recommendations. NIAID EAACI 2010 and 2013 guidelines (food allergies), BTS/SIGN and GINA 2012 (asthma), AIR 2013 (rhinitis) and SIGN 2011 (atopic dermatitis) do not recommend the supplementation of ω-3 among preventive measures because of inconsistent results in the studies so far conducted.

**Analysis of the evidence**

A systematic review (SR) published in 2014 and three subsequent studies at the closing date of the literature search of the SR was included [87–89]. De Silva’s SR includes Anandan 2009 SR and shows a prevention effect and an increased risk of developing asthma, rhinitis and atopic dermatitis [88] for ω-3 and ω-6 supplementation. No preventive effect of polyunsaturated fatty acids on the development of food allergies and other atopic diseases has been demonstrated, whether administered to children or mothers during pregnancy or breastfeeding [76, 85, 86]. Palmer and D'Vaz’s studies are randomised controlled trials evaluating the effectiveness of the preventive administration of fish oil, respectively in pregnant women (from week 21 to delivery) and children at risk. They include some serious methodological errors (such as unconfirmed diagnosis using a gold-standard test, duration of follow-up limited to the first 12 months of life, loss to follow-up > 20 % (in D'Vaz’s study) [87–89]. The results did not find statistically significant differences between the treatment group and controls. Numerous studies have also evaluated the intake of vitamins (Vit A, E, C) and minerals (Mg, Zn, Ca, P) [please note that except for this chapter the data on vitamin D is treated in a special section], but these often include methodological errors, starting with the difficulty of finding a correct quantification of dietary intakes of various foods. This makes assessing a possible relationship with allergic disease development difficult.

The results of these studies, even for the foods they investigate, are contradictory.

West et al. evaluate the effect of the administration of antioxidants (β-carotene, vitamin C, vitamin E, copper and zinc) on the development of allergic disease in children [93]. Their results demonstrate a protective effect of vitamin C on the incidence of wheezing and a preventive effect of the contribution of copper on the development of various allergic diseases. However these results cannot be used because this study has important methodological flaws.

**Recommendation. No preventive effect on the development of allergic disease of the supplementation of ω3 polyunsaturated fatty acids, vitamins or minerals has been demonstrated when administered to children and/or their mother during pregnancy or breastfeeding.**

**Box 3: Essential fatty acids**

**Table:** 

Essential fatty acids are precursors of LCP, the synthesis of which is achieved through sequential enzymatic reactions of chain elongation (elongase) and desaturation (desaturase). The enzymes involved in the reactions of elongation and desaturation of these fatty acids are common to the two biosynthetic pathways (ω-3 and ω-6 series) and display a “competing for substrate” mechanism (Fig. 1). Eicosapentaenoic acid (EPA, C20: 5 ω-3) and docosahexaenoic acid (DHA, C22: 6 ω-3) are derived from acid α-linolenic acid and arachidonic acid (AA, C20: 4 ω-6). is derived from linoleic acid. Eicosapentaenoic acid (ω-3), docosahexaenoic acid (ω-3) and arachidonic acid (ω-6) have particular biological significance as fundamental components of cell membranes (especially in the brain and retina). They are precursors of eicosanoids, compounds which consist of 20 carbon atoms (from the Greek éikosi), and are highly bioactive, acting as intercellular mediators and/or locally-acting and controlling hormones. Arachidonic acid it is the predominant precursor, present in high concentrations in all membrane phospholipids. Three different enzyme systems oxidize AA: cyclooxygenase (with training prostaglandins and thromboxanes), lipoxygenase (with the production of leukotrienes) and cytochrome P450 monooxygenase (forming 19- and 20-HETE). The biological activities of eicosanoids are varied: for example, prostaglandin E2 (PGE2) acts on blood vessels, airways, the stomach, the kidneys, neutrophil function, lymphocytes and pain receptors. Even ω-3 fatty acids can be used for the synthesis of eicosanoids, which, however, have opposed characteristics from AA-derived eicosanoids, determining smooth muscle relaxation and vasodilatation (prostacyclins). For this reason, the mechanism of competition for substrate has a very precise meaning: ω-6 fatty acids, in fact, are much more abundant in nature and in the “Western” diet, but enzymatic chain processing favours ω-3 fatty acids when present. Long-chain polyunsaturated fatty acids are best known for their possible effects on the central nervous system as a component of cell membranes, but they are also associated with the modulation of immune responses as precursors of eicosanoids [90]. The “anti-inflammatory” effect of EPA and DHA (found in fish and, in particular, their oils) is based on a competitive mechanism: at the membrane level, these LCPUFA of the ω-3 series replace AA (from which highly inflammatory eicosanoids are derived) and determines decidedly bland inflammatory eicosanoid activities. The intake of fish oil would, therefore, exert a potentially anti-inflammatory effect. In contrast, the increased consumption of vegetable oils rich in ω-6 polyunsaturated fatty acids is one of the main dietary factors in the allergic epidemic [93–95].	

**Early community exposure**

**Question 6. Should early community exposure (under 24 months of age) be recommended or not recommended for the prevention of allergic disease?**

**Introduction**

The hygiene hypothesis

In the late 1980s, the so-called hygiene hypothesis was formulated in order to explain the increased prevalence of allergic diseases observed in many epidemiological studies. This hypothesis assumed a causal association between the reduced frequency of infections and the increase in allergic disease, as a consequence of improved hygienic conditions, antibiotic prophylaxis and the widespread use of vaccination. A first important paper in support of this hypothesis in 1989 was written by David Strachan who had observed an inverse relationship between the number of siblings, birth order and hay fever in a large British cohort [97]. The immune system of children, leading a “Western” lifestyle were predominantly directed towards an allergic-type response because they did not contribute to defence against infectious agents and thus induced the maturation of a mainly T-helper type 2 (Th2) rather than T-helper type 1 (Th1) immune response. Indeed, the imbalance of T helper lymphocyte response to common environmental antigens plays a key role in the pathogenesis of atopic diseases in genetically predisposed individuals.

**Box 4: The “classical” Th1/Th2 dichotomy and the Th17 line.**

**Table:** 

The classic paradigm of the Th1/Th2 dichotomy has been made obsolete by the discovery of other cell lines. There are CD4 + T-helper cells expressing interleukin 17 (IL-17) and numerous categories of regulatory T cells (Treg) able to control effector T-cell responses have been described. While, strictly speaking, Tregs cells originate directly from thymic precursors, inducible Treg cells (iTreg) cells and Tr1 cells Th3 differ from precursors of peripheral T-helper cells through the action of several cytokines such as TG F-β, IL-2 and retinoic acid (Fig. 1). Th17 cells play a role in the immune response to self and, along the two “classic” Th1 and Th2 cell lines and Treg, represent not only a key component of the innate immune response to infection, but also exert a pro-inflammatory effect and promote tissue damage in various chronic inflammatory diseases such as asthma [98]. Recent studies have confirmed a role of this cell line in the pathogenesis of asthma in children of school age [98, 99].	

Other evidence for the hygiene hypothesis consisted in the observation of a reduced rate of allergies among children living in countries with low sanitary conditions and lower health education (associated with an increased incidence among immigrants from these countries in the West) or amid children who have contracted tuberculosis or measles, of an inverse correlation between risk of developing allergy and family educational level, as well as vaccination for measles or conditions conducive to infections, such as a large number of siblings and early age at first exposure to community contacts [97].

**State of knowledge**

Early community exposure as a protection factor in allergy development.

Early observational studies dating back to the 1990s have reported conflicting results about early exposure to the community, which lay children open to an increased risk of infections in their first year of life [100–104]. Among these, a large study including over 2000 children was conducted in Germany by Kramer et al. showing that day-care attendance between 6 and 11 months and between 12 and 24 months could be a protective factor in the later development of asthma, allergic rhinitis and skin sensitisation, limitatively to the subgroup of children without sibling [105].

Outcome variability from study to study as well as different definitions of wheezing and asthma make interpreting the data difficult. The European Respiratory Society definitions of asthma and wheezing are reported in Box 5 and are considered for the purpose of this Consensus [106]. Therefore, the results of studies using different definitions have been critically evaluated.

**Box 5: Definition of wheezing according to the definitions of the European Respiratory Society** [107]

**Table:** 

**Temporal pattern of wheezing**	
*Episodic (viral)*	Wheezing during discrete periods of time, often in association with clinical evidence of viral cold, in the absence of wheezing in the inter-critical periods
*Multiple-trigger*	Wheezing with exacerbations over time, but also symptoms during periods between one episode and the other
**Duration of wheezing**	
*Transitory*	Symptoms that begin before 3 years and disappeared (retrospectively) within 6 years; wheezing transition can be either episodic or multiple-triggered
*Persistent*	Symptoms that persisted (retrospectively) over 6 years; persistent wheezing can be either episodic or multiple-triggered
*Late-onset*	Symptoms begin after 3 years of age; either episodic or multiple-triggered

Wheezing is common in preschool children. Population studies have shown that about a third of children in their first 3 years of life develop at least one episode of wheezing which reaches a 50 % prevalence within 6 years. Wheezing is the main clinical expression of asthma and is a sign not specifically caused by the passage of air through constricted airways.

This phenomenon is often transient and resolves in most cases early, between 3 and 6 years of age, or sometimes later in childhood, between the 11th and 13th year. In a group of patients, it tends to persist into adulthood, and associates with allergic sensitisation and asthma.

Ball and colleagues evaluated the incidence of asthma (defined as the presence of at least one episode of asthma diagnosed by a physician between 6 and 13 years of age) and the prevalence of recurrent wheezing (defined by the presence of more than 3 episodes of wheezing in the previous year) in relation to the number of siblings living together and early community exposure in a large cohort of 1035 American children followed from birth. The presence of one or more older siblings living together resulted protective against the subsequent development of asthma (adjusted relative risk [aRR] for each brother: 0.8; 95 % CI: 0.7–1.0; *p* = 0, 04), as well as entry in the community during the first 6 months of life (aRR: 0.4; 95 % CI: 0.2–1.0; *p* = 0.04). Children with greater exposure to other children, at home or in day care had a higher probability of recurrent wheezing at the age of two than children without siblings or those who were not exposed early to community influences (aRR: 1.4; 95 % CI: 1,1–1.8; *p* = 0.01). These children ran a lower risk of asthma at 6 (aRR: 0.8; 95 % CI: 0.6–1.0; *p* = 0.03) and 13 years (aRR: 0, 3; 95 % CI: 0.2–0.5; *p* <0.001). However, the definitions of asthma and wheezing adopted by this last study are not those of the present Consensus. This study was also later criticized because early community exposure not only determines a greater frequency of infections but also exposes the child to other potential risk factors for asthma [108].

Other authors analysed the results by distinguishing between the different phenotypes of wheezing in accordance with the definitions of the European Respiratory Society as reported in this Consensus (Box 5). The study by Caudri and colleagues, for example, included more than 2700 children followed from 3 months to 8 years of life [109]. Risk factors significantly associated with transient wheezing were male gender, positive maternal and paternal family history of allergy, lower maternal age at delivery, high maternal body mass index, premature birth, maternal smoking during pregnancy, presence of older live-in siblings and day care attendance. Male gender, family history of allergy (maternal or paternal), no breastfeeding or lasting less than 12 weeks were identified as risk factors for persistent wheezing in this population.

Day care attendance was associated with transient wheezing, and most likely, but not certainly, may be associated with allergic asthma, also in accordance with the criteria of the modified Asthma Predictive Index (Box 6) [107, 110]. The interpretation of the results is affected by the outcomes analysed (risk factors for transient or persistent wheezing and allergic asthma, the subject of this Consensus).

Finally, infants with older siblings, those who attended childcare by 6 months of age and those with dogs at home were less likely to develop food allergy (adjusted OR [aOR] 0.7, 95 % CI 0.5, 0.8, aOR 0.5, 95 % CI 0.3, 0.8 and aOR 0.6, 95 % CI 0.5, 0.8, respectively), but these results need to be confirmed.

**Box 6: Risk factors for the development of asthma (modified Asthma Predictive Index or API) for children with wheezing (modified from reference** [110]**).**

**Table:** 

≥4 episodes wheezing last year associated with:	One major criterion: •One parent with asthma •Atopic dermatitis •Sensitisation to inhalant allergen or Two minor criteria: •Sensitisation to foods •Wheezing outside of infective episode •Eosinophilia (>4 %)
A positive API index is associated with an increased probability of developing asthma between 6 and 13 years from 4- to 10-fold. More than 95 % of children with negative API score in the first 3 years of life do not develop asthma between 6 and 13 years.	

Over the years, it seems that the consensus on the hygiene hypothesis has declined. A protective effect in allergy has been suggested for some respiratory viruses, measles, hepatitis A and tuberculosis although extensive and rigorous studies have not borne it out [110]. Other studies have reported that anti-tuberculosis, diphtheria, pertussis and measles vaccination exerted no protective effect on the development of allergic diseases before school age [111]. Some recent data have demonstrated that there was no association between the prevalence of infection in children, prevalence of infection among siblings or the use of antibiotics and the development of allergy after 2 years of life. In a Swedish study by Hagerhed-Engman and co-workers of 10,000 children, it was shown that day care attendance was not a protective factor in the development of allergy at the age of 6 years [112]. A recent systematic review by Marrs et al. could identify only a single high- quality study that investigated the association between early introduction in the community and food allergy [112, 113]. This study reported that children introduced into community exposure within the first 6 months of their life ran a significantly higher risk of sensitisation to egg, sesame and peanuts (aOR 0.5; 95 % CI 0.3–0.8) compared with children who did not attend day care [113].

Early community exposure as risk factor for the development of allergies

In contrast to the hygiene hypothesis, some studies have demonstrated that infections may instead promote the development of allergies, rather than reduce their risk. An association between viral infection (rhinovirus and respiratory syncytial virus [RSV], primarily) in early infancy and subsequent risk of asthma has in fact been reported among children at high risk [114, 115]. However, the exact role of RSV remains controversial in this setting [116]. Respiratory infections, particularly infections caused by viruses, and day care attendance could therefore constitute risk rather than protective factors for the manifestation of an atopic phenotype. The association between respiratory infections contracted at an early age and the development of allergic asthma in later periods is not yet clear, nor is whether it is secondary to the fact that children predisposed to develop allergy have a dysregulated immune system and an increased susceptibility to infection [117]. A possible association between the development of allergy and the use of antibiotics and antipyretics is also reported. However, this association may be an indirect marker of infection rather than a consequence of the direct action of these drugs [107].

A cross-sectional survey of a large Finnish population by Paunio et al., observed a higher association between measles in children and the subsequent development of allergic diseases [114]. Further, infants who spend their first night of life in the nursery have a higher risk of developing allergy than those who sleep in contact with their mother [115]. A study of over 3000 children in day care conducted in Germany by Cramer and colleagues showed day care attendance is the only risk factor from among 11 possible factors analysed for the development of atopic dermatitis within 2 years (OR: 1.56; 95 % CI: 1.31–1.86). The authors concluded that other environmental factors, not included in the hygiene hypothesis could account for this result [116].

**Conclusion**

The discussion about the validity of the hygiene hypothesis is ongoing but data from the literature and expert opinions are mixed. Early entry of a child in the community cannot currently be safely demonstrated to prevent the development of allergic disease. In contrast, some non-conclusive data suggest that infections can promote the development of allergies in children at high risk of atopy. However, further studies are needed to clarify whether this association is real or does not indicate a higher susceptibility to infections among allergic children.

Introduction

The literature data do not support the hygiene hypothesis (according to which early community exposure would protect from the subsequent development of allergies as a result of infections).

**Recommendation. Early community exposure is not recommended to prevent the development of allergy**

Introduction

Other authors, however, have suggested that early community exposure may be associated with subsequent development of allergies because viral infections contracted in the first 2 years of life could cause immune and structural changes in the respiratory tract. The evidence from the literature, however, remains controversial and therefore, does not support definite conclusions. This association could only reflect a higher predisposition of allergic children to infections.

**RecommendationEarly entry into the community cannot be considered a risk factor for the development of allergy.**

**Vitamin D**

The purpose of this document is to consider the administration of vitamin D for the primary prevention of allergies on the basis of the available scientific evidence. The targets of this analysis are paediatricians, patients’ families and all health professionals interested in allergy prevention in children.

**Introduction**

The prevalence of allergic diseases in children is affected by family history and ranges from 10 % among children with negative history to up to 20–30 % in the presence of a first-degree allergic relative [118].

In recent years a possible role in prevention has been attributed to vitamin D. Indeed, observational and cohort studies have documented the existence of a correlation between vitamin D intake (from dietary sources or supplementation) in pregnant women and children and the risk of wheezing, asthma or food allergy later in life. In the literature, epidemiological studies show that the incidence of allergic diseases also increases with increasing latitude and in consequence of decreased exposure to sunlight.

Notes on vitamin D metabolism

Exposure to sunlight is the main source of vitamin D: ultraviolet B rays induce the production of cholecalciferol (vitamin D3) in the skin, and it is then hydroxylated to 25-hydroxyvitamin D3 (25 [OH] D) in the liver. PTH then regulates the hydroxylation of 25 [OH] D to its biologically active form (1,25[OH]2D3) in the kidney. 1,25[OH]2D3 is activated by binding to the vitamin D receptor and with subsequent regulation of gene expression. Other sources of vitamin D are foods, especially fatty fish and dairy products, and administered supplements.

There is currently no common definition of the threshold below which vitamin D deficiency begins. While a 20 ng/mL threshold is generally agreed upon for musculoskeletal pathology to develop, discriminating thresholds for other health effects have not been defined. However, several studies report suboptimal vitamin D status in large parts of the population of children and adults. In Italy, recent studies have documented vitamin D levels below 20 ng/mL in 32 % of children of normal weight and in 44 % of obese children [119]. Low levels of vitamin D have been detected in up to 40 % of adolescents in one study population [120].

**State of knowledge**

A recent meta-analysis investigated the relationship between alterations of vitamin D status and a number of pathological conditions [121]. Historically, vitamin D has been associated with musculoskeletal diseases such as rachitism, osteoporosis, fractures and muscle weakness. In the last 15 years, other conditions such as cancer as well as cardiovascular, metabolic, infectious, autoimmune and allergic diseases have all been linked to rachitism.

The results of this meta-analysis supports a correlation between low vitamin D status and diseases such as hypertension, rachitism in children, vaginosis in pregnancy, the level of activity of rheumatoid arthritis, colorectal cancer and falls in the elderly. However, no conclusive evidence was found for 70 other conditions assessed.

We performed an additional search of the literature using the search strategy reported in the Appendix.

The following possible outcomes of intervention were considered:

- Atopic dermatitis
- Allergic rhinitis
- Asthma
- Food Allergy
- Allergy (any)

**Question 7. Is the administration of vitamin D recommended during pregnancy to prevent allergic diseases in children?**

**Question 8. Is the administration of vitamin D in doses higher than recommended intakes for the first year of life recommended for the prevention of allergic diseases?**

**Question 9. Is the administration of vitamin D in doses compliant with recommended intakes suggested for the first year of life recommended for the prevention of allergic disease?**

In 2012, Paul et al. evaluated 10 observational studies (including 7 neonatal cohorts) on the maternal action of vitamin D in the diet or serum levels and asthma [122]. The study conclusions were that there was not sufficient evidence to establish a causal connection; there were also no randomised clinical trial (RCT) on the effect of supplementation of vitamin D and risk of asthma. Other observational studies, including two neonatal cohorts, were subsequently published [123, 124]. Overall, 6 cohorts of newborns enrolled more than 750 infants. One showed no correlation and the other 5 found an inverse relationship between vitamin D intake (through diet or supplementation) or vitamin D levels in umbilical cord blood and the incidence of asthma or wheezing at 1–3 or 5 years of age. All these cohorts suffer from a patient dropout rate varying between 24 and 52 % and are therefore at risk of substantial bias. In the cohort studied by Rothers and colleagues, high and low vitamin D levels are associated with an increased risk of sensitisation [123]. In 2013, a randomised, controlled study by Goldring and co-workers studied 113 children receiving prenatal supplementation of vitamin D. No effect on atopy, risk of atopic dermatitis, pulmonary function and exhaled nitric oxide was found [125].

Despite a thorough analysis of the literature, in 2013 Peroni et al. published an exhaustive review concluding that there were no conclusive data identifying a possible dose of vitamin D resulting in prevention of food allergy [126].

In particular, in addition to studies showing an inverse relationship between vitamin D and atopic dermatitis, studies report that high vitamin D levels in umbilical cord blood determines a higher risk of allergic sensitisation and food allergy [127, 128].

The relationship between vitamin D and prevention of allergic diseases is strong when epidemiological and ecological studies are considered. The results remain controversial even when correlations between vitamin D levels in maternal and umbilical cord blood and allergic disease risk are measured directly. Of note, the then only available RCT (Goldring) did not show any such correlation.

**Conclusions**

Although the available literature data suggest the existence of a relationship between latitude and its consequent degree of ultraviolet radiation exposure, vitamin D levels and the prevalence and severity of allergic diseases, the data of observational studies are encouraging, but controversial and the only available RCT fails to show an effect on allergic disease prevalence.

The use of vitamin D supplementation for the primary prevention of allergic diseases remains an attractive topic of study, but current knowledge and available evidence does not warrant its recommendation [129].

Several randomised controlled trials are underway and they will certainly inform future recommendations on the basis of more solid evidence.

**Recommendation. On basis of the available evidence, the use of vitamin D supplementation is not recommended for the primary prevention of allergic disease.**

**Probiotics and prebiotics**

**Question 10. Should probiotics or prebiotics be administered to women during pregnancy for the purpose of prevention of allergic disease in their children?**

**Question 11. Should probiotics or prebiotics be administered to women during breastfeeding for the purpose of prevention of allergic disease in their children?**

**Question 12. Should probiotics or prebiotics be given to (exclusively and otherwise) breastfed infants for the purpose of preventing allergic disease?**

**Introduction**

This aim of this section is to consider the use of probiotics and prebiotics for the primary prevention of allergies on the basis of the available scientific evidence. In recent years, alterations in the intestinal microbiota have been considered as a factor for the modulating of immune and inflammatory responses, and these changes have been indicated as a possible cause of the increase in the incidence of allergic diseases [130, 131]. This is a consequence of the theoretical concept that changes in the opposite direction may be able to reduce the risk of developing allergic disease. These considerations are the basis of the hygiene hypothesis as exposed below.

**The hygiene hypothesis**

The immune system at birth has some knowledge of the self, but little experience of the outside world, and what it has been transferred through the placenta from the mother.

After birth, the development of the immune system necessitates contact with microorganisms, so that it can properly develop some basic functions: among them, the acquisition of specific memory for molecular patterns which later accelerate the subsequent recognition of potential pathogens, the maintenance of a level activation of innate immunity and the support of the development of regulatory mechanisms that block, via a Treg-mediated response, the onset of autoimmune and allergic diseases.

The incidence of autoimmune and allergic diseases increases if this mechanism is lacking. Interactions with numerous micro-organisms such as fungi, bacteria, protozoa, helminths and bacteria of the human microbiota (in the intestine, on the skin or in the respiratory and urogenital tracts) have been incorporated in the genesis and maintenance of mechanisms of immune regulation. An evolutionary concept of the hygiene hypothesis has then been developed. This concept focuses on lifestyle changes that can reduce overall exposure (and therefore beneficial interactions) between the immune system and these immune regulatory agents. In particular, because of this western lifestyle many of these exposures are increasingly reduced (agricultural methods resulting in low biodiversity in food crops, reduced exposure to ecto- and endo-parasites, reduced prevalence of chronic infections) [132]. In this context, therefore, we are particularly dependent on our human microbiota which this has an important role to play as an immunoregulatory factor. Even so, the microbiota undergoes a process of quantitative and qualitative reduction. In many studies, this process is associated with an increased incidence of allergic and autoimmune diseases, which result from a lower efficiency of the regulatory functions exercised by the immune system. The experimental model of supplementation with prebiotics and/or probiotics is based on these findings. This model aims towards inducing and maintaining a high bacterial load and a qualitative richness of the gut microbiome to subsequently correct the effect of reduced exposure linked to lifestyle.

**Probiotics**

Probiotics are ubiquitous in the daily diet of all humans. This document considers the administration of probiotics as supplements but it does not consider the exposure that occurs with common food intakes that may naturally contain probiotics (yogurt, fermented milk and the like). From a methodological point of view, the following populations are considered for possible intervention with probiotics: pregnant women, breastfeeding mothers, infants exclusively and not exclusively breastfed.

All types of probiotics and doses thereof were considered. Studies in which probiotics were used for preventive purposes were evaluated.

The following possible outcomes of intervention were considered:

- Dermatitis
- Allergic rhinitis
- Asthma
- Food Allergy
- Allergy (any)
- Adverse events
- Nutritional status

**Results**

In this consensus, we considered the results of a meta-analysis in the literature and we integrated it with the consultation of the literature and EAACI guidelines on the primary prevention of food allergy [133].

**Analysis of knowledge**

Previous recommendations. We first searched in literature for the presence of recommendations on each question.

**Question 10**

The recommendations for supplementation in pregnant women are poor: the guideline of the Finnish Medical Society recommended the administration of probiotics for primary prevention of allergies but only 2 studies (included in the subsequent metanalysis) were considered. The NIAID guidelines do not make specific recommendations. The 2006 NASPGHAN Report and the 2007 and 2011 Cochrane Database reviews do not provide guidance and report uncertain evidence (paediatric studies only) [134, 135].

**Question 11**

The recommendations for the supplementation of mothers during breastfeeding are poor: the Finnish Medical Society guidelines do not recommend the administration of probiotics for the primary prevention of allergy in mothers during lactation, but only two studies (included in the subsequent metanalysis) are considered. The NIAID guidelines do not make specific recommendations. The 2006 NASPGHAN Report and the 2007 and 2011 Cochrane Database reviews do not provide indication and report uncertain evidence (paediatric studies only) [134, 135].

**Question 12**

The recommendations in favour of supplementation are poor: the guidelines of the Finnish Medical Society recommend administering probiotics for primary prevention of allergy, but only 2 studies (included in the subsequent metanalysis) are considered. The NIAID guidelines do not make specific recommendations. The 2006 NASPGHAN Report assigns a level-I evidence rating and the 2007 and 2011 Cochrane reviews do not provide indication and report uncertain evidence (paediatric studies only) [134, 135].

**Question 10**

We evaluated 8 systematic reviews on this question published between 2007 and 2013. Their main targets are atopic dermatitis (five studies), asthma/wheezing (two) and safety (one).

Atopic dermatitis

The meta-analysis by Lee et al. analysed data from 1581 patients for pre- and post-natal administration and shows a preventive effect with a RR of 0.69 (CI: 0.57–0.83) [136]. Betsi and colleagues analysed the data of 4 studies [136]. Three studies (584 patients) reported a significant reduction in the incidence of dermatitis and one study (89 patients) did not detect any preventive effect. Doege and co-workers’ meta-analysis also documented a modest preventive effect (RR: 0.82, CI: 0.71–0.95; 2843 patients) through the administration of *Lactobacilli*, but this did not into a similar an effect when mixtures of probiotics were used [137, 138]. Lint et al. reported data extracted from a meta-analysis of 13 studies and found a significant preventive effect (RR: 0.79, CI: 0.71–0.88) [139]. A specific subgroup analysis did not distinguish between specific strains (a sub-analysis of six studies with *Lactobacillus rhamnosus* GG showed comparable efficacy) nor for routes of administration (pregnant mother, mother/nursling or child). In Foolad and colleagues’ systematic review of 9 of 10 studies, a reduced risk of atopic dermatitis was reported with estimated efficacy varying from 30 to 70 % [140].

Other allergic diseases

Supplementation with probiotics was not protective against food allergy, asthma or allergic rhinitis in either metanalysis [141, 142].

Safety

Dugoua et al.’s meta-analysis failed to detect any maternal side effect following administration of *Lactobacilli* and *Bifidobacteria* [143].

An assessment of adverse effects across all age groups considered 622 studies. Only in 387 studies were adverse events adequately reported and differences in gastrointestinal symptoms (RR 1.00 [0.93–1.07]), infections or other adverse events (RR 1.06 [0.97–1.16]) associated with the use of probiotics were not detected. Adverse events with long-term supplementation remain unknown [144].

**Question 11**

No examined systematic review directly address this question and thus the evidence needed to answer this issue must be derived from the studies previously described.

**Question 12**

The method of administration (for children only) was evaluated by some revised versions of the studies mentioned and in part already displayed [134–136, 142, 143, 145, 146]. Only Osborn’s meta-analysis detects an effect on the prevention of atopic dermatitis, but heterogeneity across studies makes the extent of such an effect uncertain. In other reviews, no efficacy of probiotic supplementation for the prevention of allergic disease has been observed.

In 2014, a systematic review once again confirmed the ineffectiveness of supplementation with probiotics for the prevention of food allergy. Experimental work conducted in a population of 220 children showed a lack of effect for the prevention of allergic disease [133].

**Prebiotics**

We considered the results of meta-analyses from the literature, integrating them with the results of the consultation of the most recent articles on this topic. The rationale behind the use of prebiotics is based on the modulation of the quantity and quality of the gut microbiome, as explained in the introductory section. The methodology, the questions and possible outcomes of intervention were identical to questions about probiotics.

**Results**

In 2013 a systematic review and 2 meta-analyses were published on the subject of supplementation with prebiotics. The systematic review, carried out by Foolad and co-workers, evaluated the effect of supplementation with prebiotics on atopic dermatitis [140]. This systematic review analysed the results of two studies for the prevention of atopic dermatitis.

The first study reported a 50 % reduction in the cumulative incidence of atopic dermatitis after 2 years in children who were given a mixture of prebiotics in the first 6 months of life [147].

The second study demonstrated a reduction in the risk of developing atopic dermatitis (HR 0.56, CI: 0.323–0.971, NNT = 25 to prevent one case of dermatitis among infants) in a group of infants fed with a formula supplemented with a mixture of prebiotics [148]. Osborn’s meta-analysis does not show efficacy in the prevention of asthma (2 studies, 226 children). Four studies (1218 infants) are analysed for atopic dermatitis and efficacy in reducing the risk of eczema is demonstrated (RR 0.68, 95 % CI: 0.48–0.97; NNT 25) [149]. Subgroup analysis does not differentiate between children for risk of disease. The authors comment that the studies differ according to type of prebiotics used, duration of administration (1–12 months) and length of observation (4–24 months).

In Srinivasjois and colleagues’ meta-analysis the complete safety of prebiotic supplementation is documented for premature infants [150].

In 2013 a further study of the protective effect of prebiotics on allergic outcomes was published. In this paper by Ivakhnenko et al. 80 infants were fed formula supplemented with prebiotics and compared to breastfed infants or infants fed with conventional formula. The incidence of atopic dermatitis in the supplemented group was significantly lower, but this paper suffers from methodological problems, including the loss of more than 30 % of the enrolled population.

**Conclusions**

This comparative assessment of the literature shows a modest efficacy of probiotics in the prevention of atopic dermatitis when administered to the mother during pregnancy and to the mother and baby during breastfeeding

Favourable effects of the administration of probiotics to breast- or formula-fed infants remain unclear when probiotics were not supplemented pre- or post-natally to their mothers.

No administration modality exerts a preventive effect on asthma, rhinitis or prevents allergies. The safety profile is excellent, and significant adverse events in the treated groups were not found in any revised literature.

Supplementation of probiotics to prevent allergic diseases cannot be recommended on the basis of the available evidence. Encouraging data on a possible reduction in the risk of atopic dermatitis should be interpreted with great caution as 25 infants have to be treated to prevent a single case of dermatitis and several studies suffer from a high percentage of patients lost to follow-up.

**Recommendation*: supplementation with probiotics***

**The administration of probiotics for the prevention of asthma, rhinitis and food allergy cannot be recommended as available studies demonstrated their ineffectiveness in the literature. The administration of probiotics to the mother during pregnancy and/or after delivery, and in together with their child during the first 6 months of life, can be considered as an intervention for the prevention of atopic dermatitis, even in the infrequent cases in which the prevailing trigger is food, among children at risk. The effect is moderate but constant across studies available in the literature.**

**Recommendation*supplementation with prebiotics***

**At the state of knowledge no recommendations of the use of prebiotics can be issued.**

**APPENDIX**

For the purpose of this report, the following definitions have been used:

Probiotics: live microorganisms that confer a health benefit to the host when administered in adequate quantity as part of the supply (FAO/WHO Expert Consultation, 2001). Prebiotics: the definition of prebiotic is reserved for non-digestible substances in the diet that selectively promote the growth and activity of one or more bacteria already present in the intestinal tract, or taken together with the prebiotic when consumed in adequate quantity.

Foods/supplements with prebiotics are defined as those foods that contain, in adequate quantity, prebiotic molecules capable of promoting the development of bacterial strains beneficial to humans.

**Indoor allergens**

**Question 13. Should environmental prevention for dust mites be recommended for children at risk of allergic disease?**

**Introduction**

Dust mites have been recognised as a major source of indoor allergens since the 1960s; *Dermatophagoides farinae* and *Dermatophagoides pteronyssinus* are the most frequent species of house dust mites found in temperate regions.

The mites feed on organic material, including flaking skin cells, fungi, yeasts and bacteria. They are made up of 75 % water and retain an optimal hydrostatic equilibrium in environmental conditions characterised by levels of relative humidity equal to 65 %. The main factor that affects mite growth is the degree of relative humidity; its reduction to below 50 % results in a reduction in the scale of their proliferation [151].

Mites release numerous allergens in the environment, including cysteine protease (*Der p* 1, *Der f* 1), serine proteases (*Der p* 3, 6 and 9), glycosidases, carbohydrate binding proteins, calcium-binding proteins, muscle proteins and cytoskeleton. Proteases, in particular, exert a pro-inflammatory activity in humans (through a non-IgE-mediated mechanism) while tropomyosin (*Der p* 10) explains the existing cross-reactivity of these proteins with others found among arthropods such as crustaceans [152].

There is evidence to suggest that exposure to dust mite allergens at levels above 2 mg/g is associated with a very high risk of developing allergic sensitisation [153]. Exposure levels greater than 10 g/g would be associated with exacerbated asthma in patients allergic to mites [154]. The reduction in exposure to these allergens, therefore, could reduce the severity of asthma and support the concept of prevention through environmental control.

**Summary of knowledge**

Which are the main environmental sources of mites?

Mites develop inside the home in conditions characterised by high levels of relative humidity which, in temperate regions, follow seasonal trends. These arthropods particularly thrive in mattresses and pillows but carpets and rugs are also excellent reservoirs for the mites [155].

Basic intervention to reduce exposure

An approach with different points of intervention against facilitating factors, sources and accumulation of mites in reservoirs is necessary to reduce exposure. Intervention is entirely empirical and optimal if living in an environment where there are no mites because of the characteristics of the microclimate in terms moisture and temperature, such as at high altitude locations (>1500 m above sea level). Reproducing these conditions at home often fails to provide the same results in terms of absolute environmental control.

Ideally, the most effective measure is to reduce relative humidity and keep it low between 35 and 50 % throughout the year irrespective of external environmental conditions.

High relative humidity actually constitutes the most important factor favouring mite growth [156]. Mattresses, pillows and sheets must be free of mite allergen, carpet, rugs and other items that can become a reservoir should be completely dispensed with [157].

In temperate climate homes, mite colonisation of mattresses ordinarily occurs within 4 months of starting use, regardless of the material of the mattress [158]. In other words, a mattress that because of its characteristics is claimed to be allergen-free does not exist. The most effective way to prevent mite colonisation is to sheathe these items in covers made of fabric that is proof to mite penetration, but also to allergens and to start using them when the mattress is still new. For mattresses and pillows that are already contaminated, the special fabric cover can trap dust mites and allergens present and prevent dispersal and contact with those who use them [157]. Not all fabrics are the same, though and these textiles which are mostly microfibers should allow the passage of air and water vapour for proper perspiration while keeping weave tension to ensure an average pore diameter blocking the passage of allergens. These pores, if of a diameter under 10 m, are able to block the passage of mite allergens, while those with a diameter less than 6 pM, also block the *Fel d* 1 allergen from cat. Fabrics that do not have these characteristics are not recommended, as well as non-washable fabrics, which accumulate allergen without the possibility of removing it. Periodic washing of mattress covers at temperatures > 60 °C allows eliminating mites anchored to the weave [157].

Intervention through ad hoc pillow and mattress covers should be extended to all pillows and mattresses (used by siblings etc.) present in the same room as the child.

A recent meta-analysis suggests that the use of anti-mite mattress and pillow covers, although effective in reducing the levels of exposure to mites, does not ensure the complete prevention of the development of allergic disease or a reduction in the extent of the symptoms [159, 160]. In other words, a single intervention does not guarantee the absolute effectiveness of environmental prophylactic measures and using a stepped, multifaceted approach that considers several aspects is probably better [161, 162]. Areas considered as allergen sources should be vacuumed regularly and thoroughly at least once a week to prevent the growth of mites, especially in homes with carpeting [163]. To be effective, vacuum cleaning should capture the particles which carry the dust mite allergens and prevent their dispersion. Suction alone does not eliminate all the mites, as they are often well anchored to textiles, but it is effective to remove faecal particles which are highly allergenic. Air intake filters (the so-called high-efficiency particulate air filters, HEPA) are regarded as essential hoovering equipment as they are able to retain allergenic particles, thereby avoiding their re-suspension and fall-out [157].

Physical measures such as freezing, heat and drying (for blankets, sheets and even toys) should theoretically be effective, as mites are killed by extremes of temperature (below −20 °C and above 60 °C). However, randomised clinical trials have yet to show a benefit of these measures. Therefore, their use can be recommended but is considered optional [157]. The use of acaricides is not recommended as their effectiveness in prevention does not warrant this type of intervention [157].

Are there specific tests to measure the load of environmental allergen?

Tests for the measurement of mite allergens in the environment are commercially available (on line “Home dust mite test kits”). These methods measure the levels of guanine and specific allergens using polyclonal and monoclonal antibodies. Currently, this approach is limited to research setting and should not be used in clinical practice.

**Conclusions**

Primary prevention of IgE-mediated sensitisation to dust mite allergens consists in avoiding persistent and complete exposure to allergen for as long as possible, especially in the early years of life. While very effective prophylaxis can be observed in regions with a dry climate and altitude in the mountainous regions (1500 m above sea level) where the mites do not survive, the complete elimination of allergen exposure in homes located in areas where mites are prevalent is difficult [164].

Even if exposure could be completely avoided within the domestic environment, intermittent exposure occurring elsewhere during the day (such as grandparents’ home, kindergarten, school or even in the street) can still lead to dust mite allergen sensitisation [165–167]. Accordingly, many attempts at primary prevention against mites cannot be effective. A correlation has been demonstrated between the extent of allergen exposure and risk level for sensitisation [168]. Therefore, the amount of exposure to mite allergens should always be reduced as much as possible [169] Table 1 and Box 7.

**Table 1:** Evidence of recommendation for anti-mite measures

Action on	Type of intervention	Level recommendation
Facilitating factors	Hygrometer	Strong
	Dehumidifier	Strong
	Central air conditioner	None
Allergenic source	Dry	Strong
	Acaricides	Not recommended
	Freezing	None
Reservoir	HEPA vacuum cleaners	Strong
	Pillowcases, mattress	Strong
	Pillowcases, pillow	Strong
	Kit for quantitative evaluation	Poor
	Denaturing agents	Not recommended

**Box 7: Recommendations for action to reduce mite allergen exposure.**

**Table:** 

- Reduce the level of relative humidity in the house, keeping it at around 50 %. Use hygrometers and, possibly, a dehumidifier; - Use pillow and mattress covers and cushion covers made of fabric labelled “antimite”; - Eliminate allergen sources/reservoirs such as carpets, rugs, curtains, stuffed animals; - Hoover periodically with a vacuum-cleaner that can retain allergen in a HEPA filter; - A multifaceted approach that includes all the previously described measures is likely to be more effective and is thus recommended; - Measures targeted at the physical elimination of mites (washing at high temperatures > 60 ° C, freezing, drying) are theoretically effective and can be recommended but clinical trials are lacking that show their effectiveness; - The use of acaricides is not recommended (limited effectiveness and possible toxicity)	

Can allergic disease be avoided?

The aim of secondary prevention is to reduce the risk of developing asthma and rhinitis in children already sensitized to mites, usually during the early years of life [170]. Several longitudinal studies have shown that allergen exposure avoidance through “environmental prophylaxis” reduces the risk of developing the disease in a dose-dependent manner, particularly if this is associated with a number of other interventions [170]. Moreover, the relationship between allergen exposure and the development of disease appears to be influenced by other contributing factors, such as contact with other allergens, irritants and pollutants, such as tobacco smoke or moulds [171].

Many scientific studies have shown the importance of environmental prophylaxis for children in whom allergic disease has already developed. A global approach contributes to more effective intervention, especially if the effect of other factors that should be reduced or eliminated is taken into account. The avoidance of dust mite allergens in subjects with allergic disease is a tertiary prevention intervention which leads to a decreased incidence of exacerbations in asthma and rhinitis, a marked improvement of symptoms, a decrease in bronchial hyper responsiveness and a reduced use of medications [157].

What messages are transmitted to the patient in terms of environmental exposure and prevention possible?

Prevention of allergic diseases by modulating exposure to dust mites requires strategy and is not limited to recommendations steps. For this reason, the following recommendations which address issues of primary, secondary and tertiary education are to be used in conjunction with the accompanying tables detailing individual components of preventive action and should be discussed with parents in order to integrate them with their values and preferences. This recommendation is issued after due consideration of the available evidence.

**Recommendations**

Introduction

Primary prevention is difficult to achieve in our latitudes as, even if environmental prophylaxis is the most thorough, intermittent exposure to allergen (also outside the home) can still cause sensitisation. Reduction of exposure (and also of intermittent exposure) decreases the odds of developing symptoms of allergic rhinitis and asthma in children already sensitised to dust mites.

Limited exposure to mite allergen improves the clinical status of sensitised children with respiratory or skin disease (atopic dermatitis).

**RecommendationPrimary prevention of mite sensitisation is possible only through a comprehensive environmental monitoring strategy that must be assessed on a case by case basis and discussed in detail with the family.**

**Exposure to pets**

**Question 14. Is it advisable to have a pet in the house for the prevention of allergic disease in children at high risk of allergy?**

Exposure to furry pets can lead to the development of specific IgE antibodies (allergic sensitisation) in susceptible individuals. This can begin a process leading to events such as allergic asthma and/or rhinitis, especially if exposure is continuous. Actually, once a sensitized individual develops allergic disease, contact with allergen triggers symptoms is often associated with poorly controlled disease. Thus, the identification of exposure sources and their removal can be considered as treatment [172]. In the case of pets, there are many and conflicting data about whether early and continued allergen exposure (especially in the first 3 months of life) may exert a protective action against sensitisation (primary prevention).

However, most studies are observational and their results are influenced by the fact that pets are more rarely kept in homes with a family history of atopy than where there is none. A recent review of longitudinal studies concluded that the relationship between exposure and risk of atopy is controversial. Results from the included cohort studies seem to indicate that exposure to dogs during childhood protects from the development of dog allergen sensitisation itself [173]. In conclusion, there is evidence that the first year of life represents a critical period where exposure to dogs or cats can affect sensitisation to pet animal allergen [174]. Although exposure to high allergen levels may theoretically reduce the risk of atopy, this reduction is not sufficient per se to recommend pet-keeping as prevention for allergic sensitisation. However, there is no recommendation to remove household animals from the home in order to prevent atopy. In secondary prevention (i.e., when a child is already sensitized) exposure should be minimised in order to eliminate the risk of progression of disease to asthma and rhinitis. In tertiary prevention, exposure to pet allergens should be minimised in order to reduce the risk of exacerbation of asthma or rhinitis symptoms. In children with an allergy clearly related to pet exposure, contact with their animal can also be minimized. Levels of intervention if the patient is already sensitised: the removal of the animal from the home is recommended to reduce the total level of exposure, even if the allergen load in the domestic environment gradually decreases, and particularly in the case of cat allergy. The characteristics of the cat or dog itself, such as hair length, sex, breed, reproductive status and the time it spends inside the home cannot be associated with environmental allergen load [175]. Data on the effectiveness of such measures of dog or cat castration are inconsistent; for these interventions have never been studied” or “for these interventions are controversial”. Recommendation for this procedure in order to reduce allergen exposure [172]. Global intervention in the primary prevention of cat or dog sensitisation is clearly preferable, but has already been shown to be controversial.

Here we only considered the clinical aspects of pet allergy in formulating these conclusions, and purposely did not address other issues, such as zoonotic risk or the emotional implications for children having to deliberately or unintentionally part with their animal. These aspects should always be taken into account when providing advice to families. Once that has been established to raise awareness, subsequent exposure can determine disease progression through exacerbation and reduced control. If removing the animal is not feasible (recommended action, ref. #1), implementing a series of measures designed to limit the level of allergen exposure should be considered [172, 176]. These measures, if implemented globally, can reduce exposure. With the animal still present at home, a series of stringent steps is required to achieve benefits [176]. These include the removal of allergen reservoirs, restraining the animal out of the house (again) or at least out of the bedroom area of the house, regularly bathing the animal [177, 178], improving home ventilation, and considering the possibility of pillow and mattress cover use for bed hygiene. There is no evidence that the use of products applied to the animal’s fur is able to reduce the allergenicity of pets.

**Recommendation. A pet is not recommended in order to prevent sensitisation to animal-derived allergens.**

**Indoor air quality and smoking**

**Question 15. Which of indoor air pollutants are the main risk factors for the development of allergies in children?**

Indoor air quality is affected by both external and internal sources of pollution. Pollutants can be the result of combustion processes (e.g., nitrogen dioxide, NO2) or may be released from building materials, furniture and commonly used home cleaning products (e.g., volatile organic compounds, VOCs). In addition, outdoor pollutants can enter homes and accumulate within confined spaces. Thus, the indoor environment makes a significant contribution to total exposure to pollutants [179, 180] and indoor environmental pollutants, tobacco smoke (environmental tobacco smoke, EU ETS) and household allergens - especially dust mites, mould and dog and cat epithelia - are the main risk factors for the development of allergy in children [181].

Significantly, Western children and adolescents spend most of their time indoors and therefore all interventions designed to reduce exposure to these environmental risk factors as much as possible are an important opportunity for prevention [182, 183]. Schools are an indoor environment of particular interest: school environments are crowded places where different types of allergens can persist for a long time. In Europe, the Health Effects of the School Environment (HESE) pilot study and the School Environment and Respiratory Health of Children (SEARCH) study evaluated the effects of pollution on the health of school children. In the HESE study, which was carried out in Sweden, Denmark, Norway, France and Italy (Siena and Udine) and included more than 600 children (mean age 10 years), a significant associations was found between exposure to > 1000 ppm CO2 concentrations and risk of dry cough, nocturnal cough and rhinitis.

Moreover, decreased nasal patency has been observed in children exposed to >50 mg/m of particulate matter (PM10) in the classroom [181]. The analysis of the data collected during the study HESE also suggested that classroom levels of mould > 300 CFU (colony forming units) per cubic meter of air exposed children to an increased risk of nocturnal dry cough [184, 185].

**RecommendationIn Western countries, children and adolescents spend most of their time in indoors: all interventions designed to reduce exposure to risk factors as much as possible should include tobacco smoke and indoor allergens (house dust mites, moulds and dog and cat epithelia in particular) represent an important opportunity for prevention.**

**Question 16. Why is exposure to second-hand smoke harmful?**

Cigarette smoking is the main source of air pollution indoors. Exposure to passive smoking in children is associated with an increased risk of several diseases, such as sudden infant death syndrome (SIDS), respiratory infections (bronchitis, bronchiolitis, pneumonia, tonsillitis and pharyngitis), adenoidal hypertrophy, impaired lung function (asthma), acute otitis media and increased severity and risk of respiratory syncytial virus infection [186]. Tobacco smoke contains more than 4000 chemical, 250 of which are known to be harmful and 50 carcinogenic. The main constituents of tobacco smoke that harm the respiratory apparatus include carbon monoxide, nitrogen oxide, formaldehyde, hydrogen cyanide, sulphur dioxide, nitrosamines, nicotine, heavy metals (lead, cadmium, nickel) and benzopyrene [187].

The effects of these chemicals are mediated by irritant, mutagenic and immunological mechanisms that are directly able to intervene in several pro-inflammatory pathways (HPr kinase, ERK1/2, JNK, nF-kB) [188]. Numerous studies have shown that many of the harmful effects of smoking are associated to its ability to interfere with the functionality of immune system cells, although the mechanism of action is not yet fully understood [189].

Passive smoking plays an immunosuppressive role by reducing Th1 cellular responses and increasing the Th2-type response, especially when exposure occurs during the first months of life [190].

There is evidence that cigarette smoking can cause alterations in both innate immunity (through reducing dendritic cell and NKC function) and the adaptive arm of immunity, (by interfering with the action of T lymphocytes) [191]. It has been shown that exposure to second-hand smoke causes a marked reduction of T-lymphocytes capable of producing IFnγ in children, thereby favouring the onset of recurrent respiratory infections [192]. It is therefore clear that passive smoking is an exacerbation risk factor in asthmatic children and is strongly linked to poor symptomatic control, higher medication consumption and reduced lung function [193].

**Recommendation. Cigarette smoking is the main source of indoor air pollution. The harmful effects of smoking are mediated by irritant, mutagenic and immunological mechanisms which promote the development of many diseases. It is essential to be aware of smoke-related diseases and to promote a smoke-free environment for children.**

**Question 17. Does exposure to passive smoking promote the development of allergic sensitisation?**

This is a hotly debated topic. The association between exposure to smoking in childhood and risk of atopic sensitisation has been extensively studied, but the literature is not univocal.

Numerous studies have been conducted to evaluate whether exposure to second-hand smoke can increase the risk of allergic sensitisation in a dose-dependent manner, especially with regard to allergens to which the child is exposed from the first months of life (dust mites, cat epithelium, food allergens).

One of the first studies of this topic showed that smoking in pregnancy is associated with increased levels of IgE in umbilical cord blood [194]. A multicenter study of 342 German children showed that a correlation existed between passive smoking and sensitisation to food allergens, but not between smoking and sensitisation to aeroallergens [195]. In 2008, Lannero and colleagues showed that early life exposure to tobacco smoke is associated with increased risk of atopy and is dose-dependent [196].

However, more recent studies offer conflicting evidence, with some finding a reduced allergic sensitisation prevalence among subjects exposed to smoke, while others reported an increased risk of sensitisation, and others still found none [197]. In view of these data, further studies are needed to clarify the causes and pathophysiological basis of the correlation between passive smoking and atopic sensitisation.

Despite the possibility that passive smoking favours the development of allergic sensitisation, it is now clear that exposure to smoke results in an extremely harmful effect on children’s respiratory function. In an extensive meta-analysis published in Pediatrics, Burke and colleagues considered 79 prospective studies and concluded that exposure to second-hand smoke causes an increase of at least 20 % in the incidence of wheezing and asthma in children [198].

**Recommendation. The association between exposure to smoking in childhood and risk of atopic sensitisation has been extensively studied, but the literature offers no consensus on this topic. However, the severity of smoke-related diseases suggest recommending absolute avoidance of exposure to second-hand smoke, regardless of the possibility that smoking may promote allergic sensitisation**.

**Question 18. What are the prevention strategies to protect children and adolescents from smoking?**

According to the World Health Organization (WHO), smoking is the leading cause of preventable deaths worldwide and is responsible for more than 5 million people dying from cancer, cardiovascular and respiratory disease every year.

More than 50 % of children are currently exposed to second-hand smoke in the home, especially in families of lower socioeconomic status. Interventions on parents and other household members have therefore a positive effect on children. Children are particularly affected by exposure to second-hand smoke because of the inadequate capacity of parents to create a smoke-free environment both at home and elsewhere [199].

Paediatricians have a fundamental role in promoting the health of children and adolescents. In regard of smoking, paediatricians are facing a twofold challenge: on the one hand they have to make caregivers aware of the dangers of exposure to second-hand smoke for children, on the other they have to inform adolescents regarding the risks associated with active smoking [199].

In order to achieve such an effective prevention strategy it is important to find out how children are exposed to smoke. In preventive medicine, we usually distinguish between first-, second- and third-hand smoking. First-hand smoking is active smoking. It might seem less relevant for paediatric patients, however, as WHO data show that adolescence is the age when most smokers develop the habit. Second-hand smoking is the so-called “passive smoking”, defined as the involuntary inhalation of substances from cigarettes, pipes or cigars smoked by other individuals.

Third-hand smoking, lastly, consists in being exposed to residues from smoking remaining in the environment, on clothes, furniture, car seats, etc. To give one example, a mother who lights up on the balcony of the house so as not to pollute the home environment will prevent direct exposure of her child to cigarette smoke, though not to third-hand smoke since residues deposited on her clothes which can be subsequently breathed in by her child.

Thus, it is important not to forget that the effects of tobacco smoke exposure are substantially identical even when caregivers smoke far away from their children and even from the domestic environment (e.g., at work, in cars, on balconies and other outdoor spaces). In consequence, it falls on the paediatrician to make families aware of all types of potential exposure to tobacco smoke. In order to reduce and, hopefully, wholly avoid smoke exposure, a multidisciplinary approach is needed and must enlist government, institutions and schools.

Intervention must focus on eliminating smoking among individuals of all ages. Primary prevention interventions are intended to reduce tobacco supply for, and access to, minors and promote motivational campaigns.

The most effective intervention is to discourage active smoking in adolescents. The main measures is targeted especially on health information and education in schools, on the prohibition of smoking in all school buildings and areas open to the public. It is important to ban cigarette advertising and sales directed at minors, to restrict adult smoking and to increase duties on cigarettes. It should be also stated the importance of referring smoker adolescens to a specific tobacco programm.

It is also worthwhile to remind caregivers that smoking cessation can ameliorate their and their children’s health. When primary prevention measures fail or a smoking habit is already formed, secondary prevention should be attempted in order to limit the smoking habit of young people [200].

It has recently been observed that the use of so-called “electronic cigarettes” has become widespread. Even if these devices are able to help adult smoker to quit smoking, little is known about their possible impact on health as passive smoking. In the absence of safety data regarding the effects of exposure to electronic cigarette’s vapour, exposure is not recommended. It is also important to recommend keeping all necessary implements for electronic cigarette smoking out of the reach of children.

In the US, cases of poisoning are on the rise as children may ingest the substances they contain [201].

**Recommendation. Paediatricians have a fundamental role to play in the promotion of all aspects of child and adolescent health. Facing the problems caused by smoking, paediatrician encounter a twofold challenge: informing caregivers about the dangers of child exposure to second-hand smoke and educating adolescents about the risks associated with active smoking.**

**Conclusion**

Numerous studies have been conducted to identify factors that trigger allergic sensitization and possible primary prevention strategies.

Sometimes they gave incontrovertible results : Consensus acknowledges them and clarifies what are the interventions that can be implemented in the high risk child.

In some cases, however, the search results are insufficient or conflicting and, translated into the clinical practice, make difficult the task of the pediatrician.

In clinical activity we must therefore always remember that he is guilty not only doesn’t do what would be useful, but also do what is unnecessary. Do what is unnecessary has a cost to society, the family and the child.

The lack of evidence of efficacy to the current state of the art does not necessarily imply that some interventions could not be effective in the future. If rigorous studies will demonstrated efficacy, these interventions would then be recommended.

Clinical work is difficult and fraught with potential errors, even when carried out according to the most rigorous scientific criteria. Outside of these criteria the clinic becomes a risky practice, unpredictable in its consequences. Disseminate and reiterate to pediatricians this general concept is an additional merit of this Consensus.

**Question 19. Why do moulds constitute an environmental risk factor for children?**

Changes in room temperature, humidity and ventilation play a fundamental role in the development and/or exacerbation of the symptoms of bronchial asthma. In particular, the exacerbation of asthma symptoms in children is commonly related to increased environmental humidity and exposure to moulds [202]. There are several species of moulds, but the most common are *Alternaria, Cladosporium, Aspergillus and Penicillium*. Among these, *Alternaria* is certainly the one which has been most studied [203].

Asthma and allergic rhinitis are strongly correlated with mould exposure during the first year of life in children and adolescents. The PATY study (Pollution and the Young), has confirmed the positive relationship between visible mould, asthma and sensitisation to inhalant allergens [204]. In the Cochrane review published by Sauni and colleagues, preventive interventions against moulds have been shown to reduce (even if only partially) the number of medical visits related to asthma exacerbation [205]. Prevention of exposure to mould has proved an important strategy to prevent allergic sensitisation, but avoidance of allergen exposure becomes crucial for the child with documented sensitisation.. The following steps must be followed for allergic patients: eliminate possible sources of moisture in basements (e.g., underground pipe leakage or seepage), dehumidifier use in damp areas of the house to keep humidity levels below 50 %, and regularly changing air-conditioner filters [206].

**Recommendation. Asthma and allergic rhinitis are strongly correlated with exposure to mould during the first year of life in children and adolescents. Prevention for exposure to mould has been demonstrated to be important in preventing allergic sensitisation. Avoidance of allergen exposure is crucial for children with documented mould allergy.**
